# AAV2-Mediated Subretinal Gene Transfer of hIFN-α Attenuates Experimental Autoimmune Uveoretinitis in Mice

**DOI:** 10.1371/journal.pone.0019542

**Published:** 2011-05-17

**Authors:** Lichun Tian, Peizeng Yang, Bo Lei, Ju Shao, Chaokui Wang, Qin Xiang, Lin Wei, Zhougui Peng, Aize Kijlstra

**Affiliations:** 1 Zhongshan Ophthalmic Center, Sun Yat-sen University, Guangzhou, People's Republic of China; 2 The First Affiliated Hospital of Chongqing Medical University, Chongqing Key Laboratory of Ophthalmology and Chongqing Eye Institute, Chongqing, People's Republic of China; 3 Eye Research Institute Maastricht, Department of Ophthalmology, University Hospital Maastricht, Maastricht, The Netherlands; University of Florida, United States of America

## Abstract

**Background:**

Recent reports show that gene therapy may provide a long-term, safe and effective intervention for human diseases. In this study, we investigated the effectiveness of adeno-associated virus 2 (AAV2) based human interferon-alpha (hIFN-α) gene therapy in experimental autoimmune uveoretinitis (EAU), a classic model for human uveitis.

**Methodology/Principal Findings:**

An AAV2 vector harboring the hIFN-α gene (AAV2.hIFN-α) was subretinally injected into B10RIII mice at two doses (1.5×10^6^ vg, 1.5×10^8^ vg). AAV2 vector encoding green fluorescent protein (AAV2.GFP) was used as a control (5×10^8^ vg). The expression of hIFN-α in homogenized eyes and serum was detected by ELISA three weeks after injection. The biodistribution of vector DNA in the injected eyes, contralateral eyes and distant organs was determined by PCR. EAU was induced by immunization with IRBP_161–180_ three weeks following vector injections, and evaluated clinically and pathologically. IRBP-specific proliferation and IL-17 expression of lymphocytes from the spleen and lymph nodes were assayed to test the influence of the subretinal delivery of AAV2.hIFN-α on the systemic immune response. hIFN-α was effectively expressed in the eyes from three weeks to three months following subretinal injection of AAV2.hIFN-α vector. DNA of AAV2.GFP was observed only in the injected eyes, but not in the distant organs or contralateral eyes. Subretinal injection of both doses significantly attenuated EAU activity clinically and histologically. For the lower dose, there was no difference concerning lymphocyte proliferation and IL-17 production among the AAV2.hIFN-α, AAV2.GFP and PBS injected mice. However, the higher dose of AAV2.hIFN-α significantly suppressed lymphocyte proliferation and IL-17 production.

**Conclusions/Significance:**

Subretinal delivery of AAV2.hIFN-α lead to an effective expression within the eye for at least three months and significantly attenuated EAU activity. AAV2.hIFN-α was shown to inhibit the systemic IRBP-specific immune response.

## Introduction

Uveitis is a common eye disease [1] and is one of the major causes of blindness worldwide [2]. It manifests either as an isolated intraocular inflammation or as a part of systemic autoimmune diseases such as Behcet's disease, systemic sarcoidosis or ankylosing spondylitis. Corticosteroids and immunosuppressive agents are commonly used for the treatment of uveitis. However, long-term application of these drugs frequently leads to numerous side effects. Furthermore, there are still a number of patients who do not respond to immunosuppressive treatment.

The introduction of biologic agents such as tumor necrosis factor-alpha (TNF-α) antibodies and interferon alpha (IFN-α) provides a new intervention regimen for patients with refractory uveitis [3], [4], [5]. IFN-α has been shown to have multiple immunoregulatory and immunosuppressive effects [6]. It helps to upregulate Tregs and inhibits IL-17-expressing cells in patients with Behcet's disease and other immune-related disorders [7], [8], [9]. Others found that it could suppress synthesis of IL-17 in both PBMCs and Th17 cells [10]. The capability of IFN-α in blocking IL-17, to some extent, is associated with the upregulation of Tregs and this may be one of the possible pathways in the treatment of autoimmunity diseases. However, potential side effects of these biologic agents have limited their use [3], [4]. The improvement of ocular gene transfer techniques and the application of viral vectors allow the therapeutic transgene to target the eye and are considered to overcome these drawbacks.

Currently, gene therapy has achieved remarkable success in human and animal models in various retinal diseases [11], [12], [13], [14]. The eye is a suitable organ for gene therapy [15] because of its following features. The small size and enclosed structure allow low dose administration to achieve a therapeutic effect. The convenient access and various routes of vector delivery can be used to target different layers in the eye [16]. In addition, many eye examination methods are currently available to monitor the treatment. Although recent studies have shown that immune responses can be generated after intraocular administration of AAV vector, this dose not necessarily to inhibit transgene expression nor dose it create retinal toxicity [17], [18].

Although AAV-mediated gene therapy of retinal disease caused by single-gene defects has been undergoing clinical trials [19], only few studies have been attempted in the EAU modal [20], [21]. We have developed a recombinant AAV2 vector containing the human IFN-α gene. After subretinal injection of the recombinant vectors into B10RIII mice, sufficient marked expression of this therapeutic molecule was associated with an attenuated development of EAU, a classic model for human uveitis.

## Materials and Methods

### Ethics Statement

This study was carried out according to the ARVO Statement for the Use of Animals in Ophthalmic and Vision Research. The protocol was approved by the Ethics Committee of the First Affiliated Hospital of Chongqing Medical University, Chongqing, China (Permit Number: 2009-201009). All surgery was performed under anesthesia, and all efforts were made to minimize animal suffering.

### Animals and Reagents

B10RIII mice were purchased from Jackson Laboratory (Bar Harbor, ME). All animals were housed under standard (specific pathogen free) conditions. Human interphotoreceptor retinoid binding protein peptide spanning amino acid residues 161–180 (IRBP_161–180_, SGIPYIISYLHPGNTILHVD) was synthesized by Shanghai Sangon Biological Engineering Technology & Services Ltd. Co. Complete Freund's adjuvant (CFA) containing 1.0 mg/ml mycobacterium tuberculosis (H37RA, ATCC 25177) was obtained from Sigma-Aldrich (St. Louis, MO).

### Vectors

**Figure 1:**
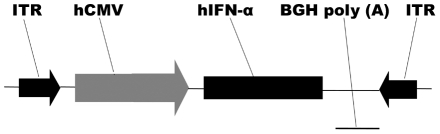
Scheme of the AAV2.hIFN-α construct. The hIFN-α coding sequence is under the control of a CMV promoter and followed by BGH poly (A). The expression cassette is flanked by ITRs. CMV promoter, human cytomegalovirus immediate early promoter; hIFN-α, human interferon-alpha; BGH poly (A), BGH poly-adenylation signal; ITR, AAV2 inverted terminal repeats.

The recombinant adeno-associated virus vector harboring human interferon alpha 2a gene (AAV2.hIFN-α) was prepared as follows. Total mRNA was extracted from freshly isolated human PBMCs using RNeasy Plus Mini Kit (QIAGEN, Valencia, CA) and first-strand cDNA was synthesized with the Superscript III Reverse Transcriptase system (Invitrogen, Carlsbad, CA, USA). The coding sequence of human interferon alpha 2a was obtained from GenBank database (http://www.ncbi.nlm.nih.gov/genbank/, GenBank accession number BC074936) and the specific primers were designed (forward, 5′ GGGGTACCATGGCCTTGACCTTTGCTTT 3′ and reverse, 5′ CTGTCGACTCATTCCTTACTTCTTAAACTTT 3′) to amplify the human IFN-α coding sequence. The PCR product was inserted into T vector (T-hIFN-α) and verified by DNA sequencing on the Applied Biosystems Model 3730 DNA Sequencing System (Invitrogen Biotechnology Co., Shanghai, China). The hIFN-α coding sequence was cut from T-hIFN-α with *Kpn*I and *Sal*I, and subcloned into an AAV2-CMV backbone between the sites of *Kpn*I and *Sal*I. After sequence verification, hIFN-α was driven by a human cytomegalovirus (CMV) intermediate-early promoter and followed by BGH poly A. The expression cassette was flanked by AAV2 inverted terminal repeats (ITRs) (Figure 1). Large-scale production and purification of vectors were performed by Vector Gene Technology Company Ltd. Vector AAV2.GFP served as a control. Titer of vectors batches were 3×10^11^ vg/ml for AAV2.hIFN-α and 1×10^12^ vg/ml for AAV2.GFP, respectively.

### Subretinal injection

Subretinal injection was performed according to the method described previously [22]. All procedures were performed under sterile conditions. Under a dissecting microscope, an aperture within the dilated pupil area was made through the cornea with a 30-gauge needle, and a blunt 33-gauge needle was inserted through the corneal opening, avoiding damage to the lens and penetrating the neuroretina. A total amount of 0.5 µl of vector suspension or PBS was slowly delivered into the subretinal space with the aid of a micro-injection system. The successful delivery of vector was confirmed by partial retinal detachment. All animals received antibiotic ointment to the cornea and were observed daily after operation. The retinal detachment resolved spontaneously. The damages occasionally induced by ocular injection included temporal corneal edema, iris-cornea adhesion or iris hemorrhage and cataract formation. The animals with any of these complications were excluded from further study.

### Human interferon-α immunoassay

Mice were sacrificed at various time points following subretinal injection of AAV2.hIFN-α. The undiluted serum was collected for assay of hIFN-α. For ocular fluid samples, AAV2.hIFN-α-injected eyes and contralateral eyes were enucleated respectively. The conjunctival tissues were carefully removed and the globes were briefly sonicated with homogenizing solution(20% glycerol, 10 mM KCl, 2 mM MgCl_2_, 0.1% Triton, 300 mM NaCl, 0.5 mM dithiothreitol, 20 mM HEPES and Anti-protease Complete TM cocktail in H_2_O, 10 µl/mg). After centrifugation at 12000×*g* for 5 min, supernatants from homogenized eyes were collected. All procedures were conducted on ice. hIFN-α concentration was determined using a VeriKine Human IFN Alpha ELISA Kit according to the manufacturer's instructions (PBL Interferon Source, USA) with a detection limit of 12.5 pg/ml.

### Vector DNA biodistribution

PCR analysis was performed to evaluate the biodistribution of rAAV2 vector DNA. Mice were sacrificed and total DNA was extracted from AAV2.GFP injected eyes, contralateral eyes and distant organs using a Qiagen DNeasy kit. Primers for GFP (forward, 5′ TGGCCCGCCTGGCATTATGC 3′; reverse, 5′ TGGAGACTTGGAAATCCCCGTGAGT 3′) and GAPDH (forward, 5′ TGACGTGCCGCCTGGAGAAA 3′; reverse, 5′AGTGTAGCCCAAGATGCCCTTCAG 3′) were used to amplify 750 bp and 98 bp fragments respectively.

### Induction and clinical assessment of EAU

Mice were immunized subcutaneously at the base of the tail and both thighs with 50 µg human IRBP_161–180_ peptide in 100 µl PBS, emulsified 1∶1 v/v in complete Freund's adjuvant (CFA) supplemented with 1.0 mg/ml *Mycobacterium tuberculosis* strain (MTB). A total of 200 µl emulsion was given for one mouse. EAU activity was examined clinically by slit lamp microscopy from day 8 to 21 after immunization. The clinical severity of ocular inflammation was assessed by two independent observers in a masked manner, and scored on a scale of 0–5 in half-point increments, according to five separate criteria described previously [23], with some modifications (table 1).

**Table 1:** Criteria of EAU Clinical Scoring in B10RIII mice.

Criteria			
**Corneal edema**	Mild	+	
	Moderate	++	Total 15 “+”
	Gross	+++	
**Conjunctival hyperemia**	Mild	+	
	Moderate	++	Grade:
	Gross	+++	0
**Ciliary injection of the cornea**	Mild	+	1 1∼3 “+”
	Moderate	++	2 4∼6 “+”
	Gross	+++	3 7∼9 “+”
**Anterior chamber inflammation**	Occasional cells present	+	4 10∼12 “+”
	Moderate or heavy cells present	++	5 13∼15 “+”
	Hypopyon or exudate in the chamber	+++	
**Posterior synechiae**	Mild (<1/4 of Posterior synechiae)	+	
	Moderate(1/4∼1/2 of Posterior synechiae)	++	
	Total seclusion of the pupil	+++	

### Histopathology

Eyes were enucleated on day 14 following IRBP immunization and were fixed in 4% buffered formaldehyde for 1 hour at room temperature. Tissues were embedded in paraffin. Serial 4–6 µm sections were cut through the papillary-optic nerve axis and stained by haematoxylin and eosin. At least four sections of each eye cut at different levels were prepared and evaluated histologically. The intensity of EAU was graded in a masked fashion on a scale of 0 to 4, as described earlier [24].

### IRBP-specific lymphocyte responses

The spleen and draining lymph nodes were removed from immunized mice on day 21. A single cell suspension was prepared by mechanical disruption and followed by a passage through a sterile stainless steel screen. For proliferation and cytokines assay, cells (2×10^6^ cells/ml) were cultured in triplicate with RPMI 1640 medium (Gibco, Grand Island, NY, USA) containing 2 mM L-glutamine, 5×10-5 M2-ME, 0.1 Mm nonessential amino acids, 1 mM sodium pyruvate and 10% FBS in the presence of 10 µg/ml IRBP_161–180_, 1 µg/ml Concanavalin A (Sigma) or medium alone for 72 hours. Proliferation was detected by a modified MTT assay using a cell counting kit (Cell Counting Kit-8; Sigma) as described previously [25]. IL-17 concentration in the supernatants was measured using a commercially available ELISA kit according to the manufacturer's directions (R&D System, Minneapolis, MN) with a detection limit of 15 pg/ml.

### Statistical analysis

Data are expressed as mean ± standard deviation (SD). Severity of EAU was analyzed using the Kruskal-Wallis test followed by the Mann-Whitney U test with Bonferroni correction. Lymphocyte proliferation and cytokine production was analyzed using ANOVA. P<0.05 was considered to be significantly different. All experiments were repeated at least twice.

## Results

### hIFN-α expression following subretinal injection of AAV2.hIFN-α vector

**Figure 2:**
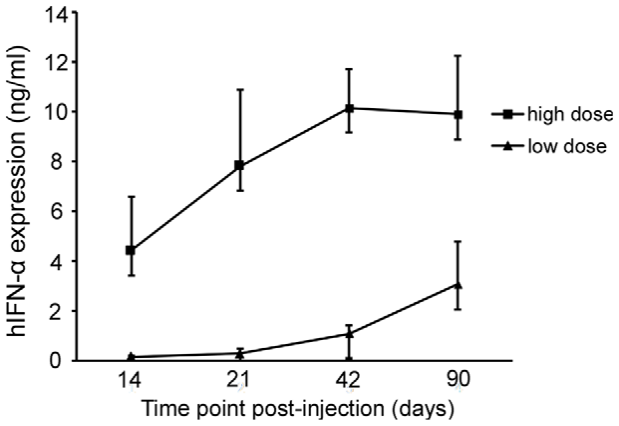
The expression of transgenes following subretinal injection of AAV2.hIFN-α. The ocular hIFN-α levels at various time points show that hIFN-α expression starts before day 14 following injection (the first time point tested). For the higher dose, hIFN-α expression reaches a peak on day 42 and remains high until day 90. In the eyes receiving a lower dose of vector, hIFN-α level shows an unremitting increase from three weeks to three months. Results are expressed as the mean ± standard deviation.

Different groups of mice were injected subretinally with two doses of AAV2.hIFN-α, 1.5×10^8^ vg or 1.5×10^6^ vg. The hIFN-α level in supernatants from homogenized eyes was assayed by ELISA. In the eye receiving a subretinal injection of the higher dose of AAV2.hIFN-α (1.5×10^8^ vg), expression of hIFN-α was detectable on day 14 (mean 4.41 ng/ml) increasing further on day 21 (mean 7.8 ng/ml). A high level of hIFN-α was observed on day 42 (10.15 ng/ml) and remained detectable until three months after injection (the last detection point). For the lower dose (1.5×10^6^ vg), the level of hIFN-α was 0.128 ng/ml on day 14, 0.25 ng/ml on day 21, sharply increased on day 42 (mean 1.067 ng/ml) and day 90 (mean 3.057 ng/ml) following subretinal injection (Figure 2). The hIFN-α expression in the low dose of AAV2.hIFN-α injected group was about thirty fold lower than that from the high dose injected group. For both doses of AAV2.hIFN-α, hIFN-α expression was undetectable in the undiluted serum or contralateral uninjected eyes over time.

### Biodistribution of vector DNA

**Figure 3:**
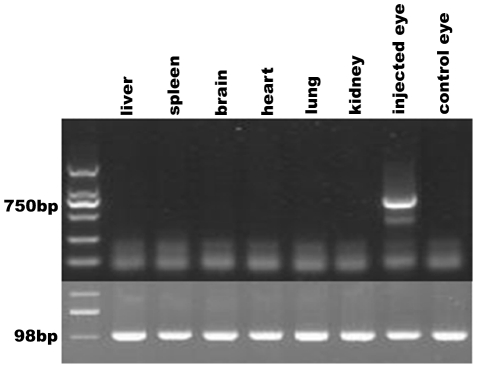
Biodistribution of vector DNA three weeks after the subretinal injection of AAV2.GFP. Vector DNA is detectable only in the injected eye, but not in the contralateral eye and distant tissues including liver, spleen, brain, heart, lung, and kidney.

PCR analysis was performed to determine the biodistribution of vector DNA after AAV2.GFP subretinal delivery. Total DNA was extracted from the injected eyes, contralateral eyes and distant organs (liver, spleen, heart, brain, lung and kidney) three weeks after subretinal injection. AAV2 vector DNA was PCR-amplified using GFP-specific primers. A 750 bp GFP-specific product was only detected in the AAV2.GFP treated eyes and no PCR product could be measured in the other tested organs or contralateral eyes (Figure 3).

### The effect of AAV2.hIFN-α on EAU

All normal B10RIII mice immunized with 50 µg human IRBP_161–180_ peptide emulsified in CFA developed EAU as evidenced by conjunctival hyperemia, ciliary injection, corneal edema, posterior synechiae, aqueous flare and cells. The inflammatory signs appeared on day 8 or 9 after immunization, reached a peak by day 12 and were followed by a gradual regression. There was no inflammation in the control mice which received CFA alone.

To test the effect of AAV2.hIFN-α on EAU, it was subretinally injected into right eyes at two doses, 1.5×10^8^ vg and 1.5×10^6^ vg. AAV2.GFP (5×10^8^ vg) was injected into the contralateral eyes as internal control [20]. Mice were immunized with IRBP_161–180_ peptide emulsified in CFA three weeks after subretinal injection. Mice receiving a subretinal injection of PBS and a subsequent immunization with IRBP_161–180_ peptide emulsified in CFA served as a separate control group.

**Figure 4:**
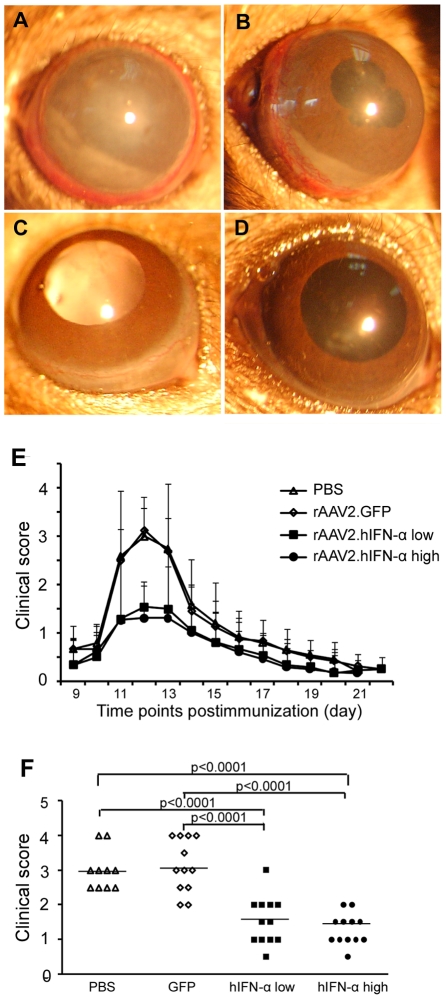
Clinical evaluation of EAU activity. Two doses of AAV2.hIFN-α were subretinally injected into the eye respectively, PBS and AAV2.GFP were used as controls. Three weeks after injection, EAU was induced by immunization with IRBP_161–180_ and ocular inflammation was examined by slit lamp microscopy. Images show significantly severe inflammation in the PBS (A) and AAV2.GFP injected eyes (B) as compared to the AAV2.hIFN-α treated eyes (C, D). Kinetics of EAU (E) reveals that subretinal injection of both doses of AAV2.hIFN-α persistently attenuated ocular inflammation of EAU as compared with PBS and AAV2.GFP. The significant difference was observed consecutively on day 11 to 14 after immunization (P<0.05). Data are presented as mean ± standard deviation. Clinical score on day 12 after immunization (F) shows that the PBS injected eyes had a score of 3 (±0.58) and the AAV2.GFP injected eyes reached a mean clinical score of 3.13 (±0.77), the score of AAV2.hIFN-α treated eyes was 1.54 (±0.69, p<0.0001, Mann-Whitney U test) in the lower dose treated group and 1.292 (±0.45, p<0.0001) in the higher dose group. Each point represents an individual eye. The average scores of each group are denoted by the horizontal bars.

**Figure 5:**
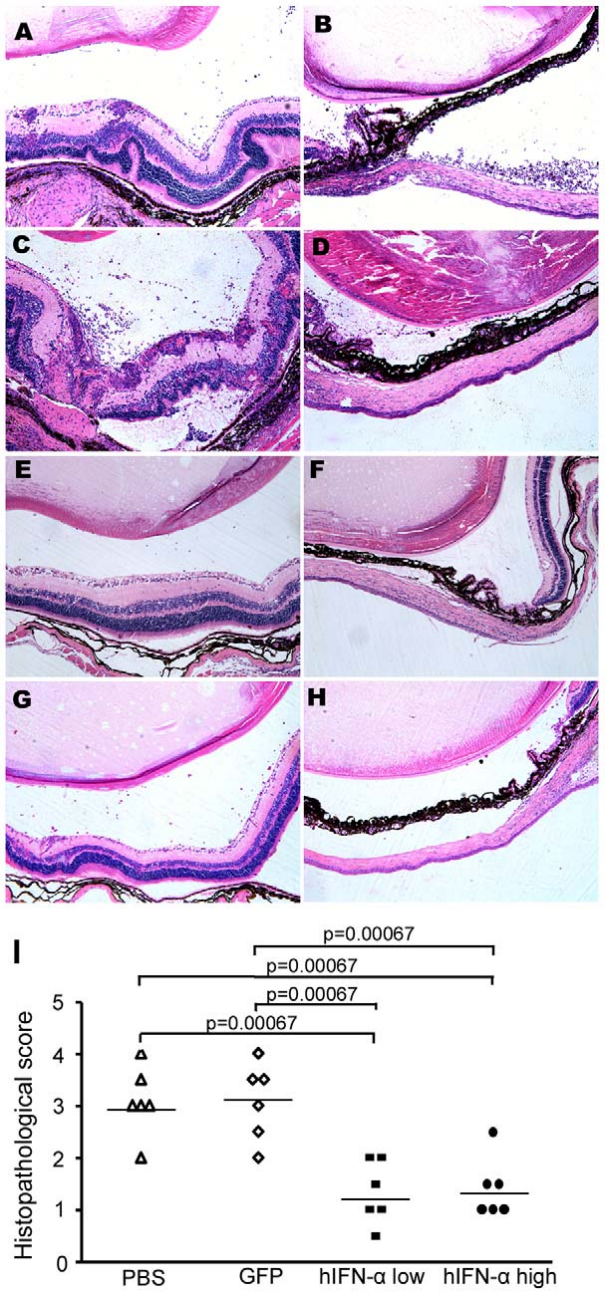
Histological examinations on day 14 of EAU. Images of histological analysis show obvious iris thickening, severe retinal folding, destruction, damage of the photoreceptor layer and massive inflammatory cell infiltration in the iris, vitreous, retina, and subretinal space, as well as intensive vasculitis formation in PBS injected eye (A, B) and AAV2.GFP injected eyes (C, D). However, a minor infiltration of inflammatory cells was observed in the vitreous and retina in both lower (E, F) and higher dose (G, H) of AAV2.hIFN-α treated eyes. (haematoxylin eosin staining, original magnification ×100) Histological grade (I) shows reduced EAU in both doses of AAV2.hIFN-α treated groups as compared with PBS injected and AAV2.GFP injected eyes (p<0.0001, Mann-Whitney U test). Each point is the score of an individual eye. The mean scores of each group are denoted by the horizontal bars.

Clinical signs were monitored after immunization by slit lamp microscopy. In PBS or AAV2.GFP injected eyes, severe uveitis, as evidenced by conjunctival hyperemia, ciliary injection, corneal edema, aqueous cells and posterior synechiae was observed (Figure 4A, B). A minor inflammatory reaction as manifested by conjunctival hyperemia or ciliary injection was found in both doses of AAV2.hIFN-α treated eyes (Figure 4C, D). Severity of inflammation was clinically scored on a scale from 0 to 5. Both doses of AAV2.hIFN-α treated eyes showed a significantly decreased activity of EAU throughout the course of disease as compared with PBS or AAV2.GFP injected controls (Figure 4E). Clinical scoring on day 12 showed that a significantly decreased severity of EAU was observed in AAV2.hIFN-α^low^ and AAV2.hIFN-α^high^ treated groups when compared with PBS and AAV2.GFP injected controls (p<0.0001). There was no significant difference between the two groups receiving different doses of AAV2.hIFN-α. Histological examination on day 14 showed severe intraocular inflammation in the AAV2.GFP injected eyes and PBS injected control mice as evidenced by massive infiltration of inflammatory cells into the iris, vitreous cavity, throughout all retinal layers and the choroid, intensive retinal vasculitis, obvious iris thickening, destruction of the retinal architecture with severe retinal folding and detachment, as well as photoreceptor damage (Figure 5A, C). However, in both doses of AAV2.hIFN-α treated eyes, only scattered infiltration of inflammatory cells into the vitreous body and retina was observed (Figure 5E, G). Additionally, the inflammatory changes in the anterior segment in both doses of AAV2.hIFN-α treated groups were less than those in the AAV2.GFP injected eyes and PBS injected mice (Figure 5B, D, F, H). Pathological grading showed that PBS injected eyes (EAU grade, 3.08±0.66) and AAV2.GFP treated eyes (EAU grade, 3.2±0.76) had significantly more intensive inflammation as compared to the AAV2.hIFN-α treated eyes (1.33±0.6 for low dose, 1.4±0.58 for high dose) (P<0.0001) (Figure 5I).

### Effects of subretinal injection of rAAV2.hIFN-α on the IRBP-specific systemic immune response

**Figure 6:**
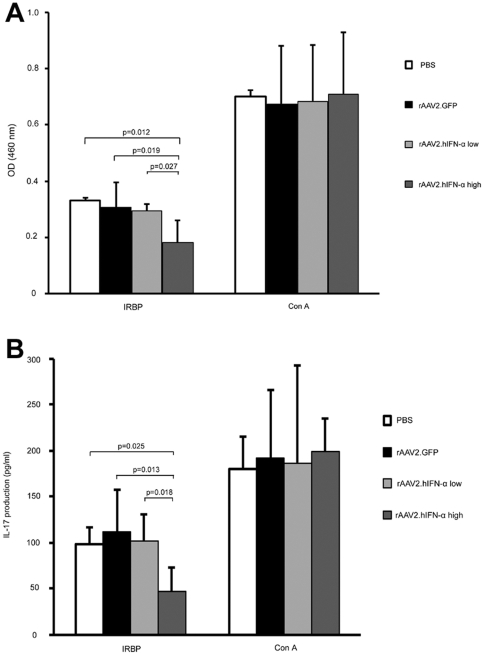
Systemic IRBP-specific immune responses in each group. IRBP-specific lymphocyte proliferation (A) and IL-17 production *in vitro* (B) show no significant difference among lower dose of AAV2.hIFN-α, PBS and AAV2.GFP treated mice (P>0.05). A significant decline of lymphocyte proliferation is observed in higher dose of AAV2.hIFN-α treated mice compared with PBS injected (p = 0.012), AAV2.GFP injected (p = 0.019) and lower dose of AAV2.hIFN-α treated mice (p = 0.027). Similarly, the IL-17 production is significantly downregulated in higher dose of AAV2.hIFN-α treated mice as compared with PBS injected (p = 0.025), AAV2.GFP injected (p = 0.013) and lower dose of AAV2.hIFN-α treated mice (p = 0.018). Results are presented as mean ± standard deviation and every experiment was performed three times.

To determine whether there was an effect of AAV2.hIFN-α on the IRBP-specific immune response, lymphocytes from spleen and lymph nodes were isolated and incubated for 72 hours in vitro with IRBP_161–180_ peptide, ConA (positive control), or medium alone (negative control) respectively. The proliferation and IL-17 production of lymphocytes were assayed. The result showed a similar response in proliferation and IL-17 production of lymphocytes incubated with ConA among the two doses of AAV2.hIFN-α treated mice, AAV2.GFP treated mice and PBS treated mice. A somewhat lower response in IL-17 production and proliferation of lymphocytes was observed in all the tested four groups when exposed to IRBP_161–180_ peptide. There was no difference concerning IRBP-specific lymphocyte proliferation and IL-17 production among lower dose of AAV2.hIFN-α treated mice, AAV2.GFP treated mice and PBS treated mice (p>0.05). However, the higher dose of AAV2.hIFN-α treated mice exhibited significantly reduced IRBP-specific lymphocyte proliferation as compared with PBS treated mice (p = 0.012), AAV2.GFP treated mice (p = 0.019) and lower dose of AAV2.hIFN-α treated mice (p = 0.027) (Figure 6A). Also, IL-17 production was significantly downregulated in the group of mice receiving the higher dose of AAV2.hIFN-αwhen compared with PBS treated mice (p = 0.025), AAV2.GFP treated mice (p = 0.013) or mice receiving the lower dose of AAV2.hIFN-α (p = 0.018) (Figure 6B). Lymphocytes from the tested four groups did not show a detectable proliferation and IL-17 production when cultured with medium alone.

## Discussion

In this study, we investigated the effect of an AAV2-based ocular gene therapy designed to make retinal cells secrete a therapeutic molecule, hIFN-α, on the development of EAU in mice. The results showed an effective expression of hIFN-α within the treated eyes following subretinal injection of AAV2.hIFN-α. The distribution of vector DNA was restricted to the injected eye without detectable spreading. Subretinal administration of AAV2.hIFN-α using either a high or low dose of vector (1.5×10^8^ vg and 1.5×10^6^ vg), both significantly reduced EAU development. The IRBP-specific immune response was not affected following the lower dose vector injection but both IRBP-specific proliferation and IL-17 production were downregulated when the higher vector dose was used.

In an attempt to achieve a successful gene therapy, an effective recombinant viral vector and a feasible gene delivery system in association with a locally effective expression of transgene without systemic spreading are all necessary. In this study we successfully prepared the recombinant viral vector encoding hIFN-α, an effective immune suppressive cytokine, based on adeno-associated virus, which has been proven to effectively transduce various ocular cell types for a long time. Subretinal injection of AAV2.hIFN-α using two doses resulted in the effective secretion of this cytokine within ocular tissues for at least 70 days. Examination by fluorescence microscopy showed that GFP was expressed by RPE cells and photoreceptors following subretinal injection of AAV2.GFP (data not shown), which are consistent with earlier reports [26], [27]. Expression of GFP within the retina suggested that hIFN-α might be secreted predominantly by RPE and cells in the photoreceptor layer. An earlier study by Lai et al [28] revealed that the secreted protein could diffuse into both the posterior and anterior segments of the eye with the natural fluid flow following subretinal injection of AAV2 vectors. It is, therefore, not surprising to note a strikingly diminished inflammatory activity both in the anterior and posterior segments in the AAV2.hIFN-α treated eyes following immunization with IRBP_161–180_ peptide. Another important factor for a successful gene therapy using viral vectors is that the administrated vector should be distributed only in the local tissue [29]. In order to examine whether the injected AAV2 vector entered into other organs, we investigated its dissemination in the contralateral eye and organs distant to the injected site. The result showed that the AAV2 vector DNA was found only in the treated eye but not in the tested organs or in the contralateral eye. This result is consistent with earlier reports in rats [30] and nonhuman primates [31] and this has been attributed to the integrity of the blood-eye barrier. However, early reports showed that AAV2 vector DNA was also detectable in the brain of intravitreally injected dogs [32] and mice [33], [34]. This result has been explained by the transport of the AAV2 vector DNA into the brain due to an extremely abundant expression of this DNA in the ganglion cells following intravitreal injection [32]. The experiments with subretinal injection of the recombinant AAV2 vector reported previously by others and the result presented here seem to avoid this unwanted distribution of the injected virus DNA into other organs. It is, therefore, reasonable to presume that subretinal injection of AAV2.hIFN-α may lead to a long-term effect of hIFN-α within the treated eye without, at least, obvious systemic biodistribution of AAV2 vector DNA.

As mentioned above, subretinal injection of AAV2.hIFN-α could lead to a therapeutic concentration of hIFN-α within the eye and was able to significantly inhibit EAU elicited by IRBP administration in the presence of CFA. We subsequently investigated whether a local administration of AAV2.hIFN-α affected the IRBP-specific systemic immune response and whether the inhibitory effect on EAU was associated with a downregulated IRBP-specific systemic immune response. As there was no tracing technique available in this experiment, we adopted a two-dose strategy to identify whether there was a difference in the IRBP-specific systemic immune response as well as an inhibitory effect on EAU following subretinal administration. Our result showed that both doses were able to significantly attenuate the EAU activity although the hIFN-α expression within the eye of the lower dose of AAV2.hIFN-α injected group was thirty-fold lower than that in the higher dose group. A downregulated lymphocyte proliferation and a decreased IL-17 production were only observed in the group of animals receiving the higher dose. However, biodistribution detection showed no dissemination of AAV2 vector DNA in systemic organs and hIFN-α in serum. Additionally, early study showed that subretinal administration of AAV2 vector did not trigger any humoral immune response [27]. Collectively, a likely explanation is that the local immunomodulatory environment in the high dose treated animals is causing local antigen presenting cells to have a less activated phenotype, so that when they migrate to the draining lymph nodes, the T-cells are not as activated and perhaps more anergic or tolerant. In view of these results, it is not likely that the inhibitory effect of hIFN-α on EAU was mediated by a downregulated systemic immune response. However, the downregulated systemic immune response, on one hand, may be useful for uveitis associated systemic diseases. On the other hand, it may lead to unknown and unwanted side effects. More studies are needed to explore and address these issues.

There are some limitations in our study. The production of hIFN-α was only followed for a time period of three months and a prolonged observation is needed to determine the duration of secretion of the transgene product after intraocular administration. Furthermore, the exact mechanisms by which the released hIFN-α exactly inhibits EAU should be explored. In addition, a more effective rAAV2 vector using a promoter modulating the expression of hIFN-α needs to be developed for future studies.

In conclusion, we have now developed an AAV2-mediated long-lasting and effective hIFN-α gene delivery system. The locally secreted hIFN-α following subretinal injection of AAV2.hIFN-α significantly reduced the activity of EAU.
